# Supplementary material for: Are carbon emissions trading and green financial instruments synergistic? -Comprehensive quantitative research based on content analysis
Source: PLoS One. 2024 Mar 7;19(3):e0298601. doi: 10.1371/journal.pone.0298601 (PMC10919601; doi:10.1371/journal.pone.0298601)
Supplement: S1 Data — (ZIP) [file pone.0298601.s001.zip › Data/地级市Z_F工作报告.zip!/╦─┤¿/┤∩╓▌╩╨/└┤╫╘╣1⁄2╓┌║┼í╛┬φ┐╦╩2╛▌═°í┐_2018.docx]

各位代表：

我代表市人民政府向大会报告工作，请予审查，并请市政协各位委员提出意见。

一、2017年工作回顾

2017年，在省委省政府和市委的坚强领导下，在市人大、市政协的监督支持下，市人民政府带领全市人民，团结一心、砥砺奋进，各项工作均取得了显著成效。预计全年实现地区生产总值1585亿元、增长8.2%，地方一般公共预算收入90.7亿元、增长10%，固定资产投资1725亿元、增长15%，社会消费品零售总额860亿元、增长13%，城镇居民人均可支配收入28250元、增长8.6%，农村居民人均可支配收入12830元、增长9.5%，居民消费价格上涨1.7%，城镇登记失业率控制在4.1%以内，单位GDP能耗同比下降2%，圆满完成了市四届人大二次会议确定的各项目标任务，为决胜同步全面小康奠定了坚实基础。

（一）经济实现快速发展。全市经济在上年止滑回升的基础上实现快速增长，GDP、规上工业增加值、地方一般公共预算收入等各项经济指标增速均高于全省平均水平且排位大幅上升。经济质量明显提高，全市税收占财政收入比重同比提高2.5个百分点，其中市本级提高5.5个百分点。“项目攻坚年”活动扎实开展，30个重大项目和200个重点项目完成投资480亿元。工业转型升级加快推进，国家天然气综合开发利用示范区获批，川渝合作产业园、玄武岩纤维产业园、云内动力专用车、宣汉正原微玻纤三期等43个重点项目开工建设，电子信息、新材料、装备制造等产业集群加快形成，新培育亿元以上企业22户，新增规上企业84户，规上工业增加值增长9.5%。服务业提档升级步伐加快，罗浮广场、红星广场、秦巴物流园区铁路物流等项目竣工投运，复兴农产品批发市场、仁和春天、巴山大峡谷和铁山旅游综合开发等项目加快建设，服务业完成投资1100亿元，旅游总收入173亿元、增长22.5%，电子商务交易额90亿元、增长20.5%，大竹县成功创建全国电子商务进农村示范县。现代农业稳步发展，粮食持续稳产，新建现代农业产业基地15万亩，新增省级示范农民合作社15个、家庭农场18家，新培育市级以上龙头企业10家，农产品加工和贸易收入达230亿元。优势潜力产业加快发展，金融机构各项存款余额突破3000亿元、贷款余额1250亿元，新增资质建筑和房地产企业63家，金融业、建筑业、房地产业增加值分别增长9.3%、11.3%和13.6%。科技创新深入开展，高新技术产业产值260亿元，科技对经济增长的贡献率达52%。“质量达州”建设全面推进，国家天然气产品、全省玄武岩纤维及制品质量监督检测中心获批。

（二）城乡面貌显著改观。综合交通枢纽建设取得新进展，机场迁建、营达高速、环城路二期、达宣和达开快速通道等骨干工程加快建设，巴万高速、机场大道等项目开工建设，成南达高铁、达州高铁站、市绕城高速西段、镇达广高速等项目前期工作加快推进，争取西渝高铁过境达州取得实质性突破，新开辟航线2条。区域中心城市加快构建，凤凰山隧道及西延线一期、金南大桥、火烽山隧道、大尖子隧道等一批标志性工程建成通车，快速通道拓宽改造、凤凰山山前路等项目加快建设，新建停车泊位1.4万个；北城滨江、西城马踏洞和莲花湖、南城三里坪和长田坝等城市新区开发加快推进，建成区面积达到90平方公里、人口超过100万，“双100”区域中心城市〔1〕框架基本形成；塔沱、火车站、川鼓等片区棚改大力度推进，大寨子、鹿鼎寨、塔沱滨水、紫荆花等公园加快建设，莲花湖湿地公园一期不断完善、二期生态修复启动实施，城市品质进一步提升；主城区环境卫生大幅改善，城市公交线网优化调整，市级云计算中心、智能交通一期工程建成投用，城市管理水平明显提高。城镇和新村建设成效显著，5个县城规模、品质、档次同步提升；百镇建设行动深入开展，宣汉南坝、达川石桥分别列入首批国家和省级特色镇，15个乡实现撤乡设镇；建设幸福美丽新村455个，建成市级“四好村” 471个。全市户籍城镇化率较上年提高1.67个百分点。

（三）改革开放成果丰硕。各项改革全面深化，“强市活区”的市区管理体制改革在探索中不断推进；城市公交管理在新的体制中高效运转；城市建管分离改革有效实施；政府绩效管理改革全面展开，单项目标考核全面取消，单位内部考核得到加强；供给侧结构性改革成效明显，140万吨钢铁去产能任务全面完成，煤炭去产能纵深推进，商品住宅去库存80万平方米；市属34户破产国企改革全面结束，2.74万名改制职工完成社保和社区双移交，10类26户国企基础改革基本完成，三大平台公司职能职责划分明晰；金融改革不断深化，达州银行首次在外设立分支机构，农村信用社改制稳步推进，直接融资突破100亿元，天府股权交易中心挂牌企业91家；商事制度改革不断加强，新登记市场主体3.3万户、增长52.7%；财政管理改革深入推进，市本级资金筹措使用统筹机制基本建立，市级综合治税平台不断完善，改革财政支持方式、清理政府经营性资产、创新政府配置资源方式全面展开；农村土地承包经营确权登记颁证基本完成；教育、卫生改革发展创新举措出台实施。开放合作成效明显，第二届达商大会成功举办，“6+2”产业集群〔2〕专题招商精准实施，小分队招商高频率开展，深圳卡立通手机及智能终端、青岛正北二硫化碳等53个项目落地建设，成功引进四川铁投建设玄武岩纤维产业园、川煤集团建设川东煤炭储配物流基地、江西正邦建设农牧产业园，与宜华集团签订建设木材交易及加工项目战略合作协议，引进北控水务技术支持城镇污水治理体系前期工作，三一重工和远大实业建设建筑产业园正在深入洽谈，全年新签约项目141个，引进到位资金768亿元；外经外贸加快发展，实际到位外资9565 万美元，完成进出口5887万美元。

（四）脱贫攻坚再战再捷。精准扶贫精准脱贫扎实推进，通川区贫困县摘帽已接受省上验收，全市311个贫困村退出、15.07万贫困人口脱贫目标全面完成，贫困发生率由上年的6.5%下降至3.7%。向上争取和市县财政筹集资金59.7亿元，集中用于住房保障、基础建设、产业发展，完成贫困户危房改造1.33万户，建成农村公路1562公里，8.4万人饮水安全问题得到解决，206个贫困村建立了电商服务站点。建成易地扶贫搬迁集中安置点154个，搬迁入住1.5万户4.8万人。圆满承办全国易地扶贫搬迁现场会，大竹易地扶贫搬迁与产业发展“双靠近三融合”〔3〕经验得到国家认可并全省推广。金融扶贫力度加大，贷款余额258亿元，居全省第2位。创新推出“5+2”帮扶机制〔4〕，828个贫困村驻村帮扶实现法律顾问、农信员全覆盖，11.4万名财政供养人员与贫困户实现结对帮扶，为贫困户超过20户的非贫困村选派了第一书记。大力开展脱贫攻坚引领区建设，连片发展主导产业10.8万亩，建设旅游景点41处，引领区内37个贫困村1.86万贫困人口率先实现退出和脱贫。

（五）人居环境大幅改善。生态文明建设持续加强，制定出台了“135”环境治理方案〔5〕，集中开展了环境突出问题整改“百日攻坚”和“年度攻坚”，市民反映强烈的达钢“白烟”等环境问题整治取得明显成效，多年污染中心城区的火车东站货场彻底关闭搬迁，主城区环卫市场化清扫保洁、建筑工地扬尘和黑臭水体治理等成效显著。积极支持配合中央和省开展环保督察，中央督察组移交的386件信访投诉问题按时按质办结上报，省环保督察发现问题整改完成率达88%。大气、水、土壤污染防治三大攻坚战深入推进，空气达标率同比上升9个百分点，PM10和PM2.5〔6〕平均浓度同比分别下降10.5%、10.7%；水和土壤环境质量保持稳定。制定实施城镇污水和城乡垃圾处理设施建设“三年攻坚”方案，市第二生活污水处理厂加快建设，全市污水处理、垃圾发电及固废处理等项目前期工作全面开展。市、县、乡、村四级河长体系全面构建，工作责任有效落实，流域治理效果明显。绿化达州行动深入开展，国家森林城市创建全面启动，自然保护区、森林公园、地质公园、湿地公园保护建设切实加强，賨人谷成功创建国家森林公园，完成营造林40.6万亩，森林覆盖率达到42.8%，治理水土流失182平方公里。

（六）民生事业全面发展。各项民生投入274.8亿元，占公共财政支出的70.9%。中心城区10大民生实事加快推进，全市九项民生工程、19件民生实事全面完成。改造棚户区及农村危房3.7万户。城镇新增就业4.1万人。全民参保计划深入实施，“五险合一”〔7〕征缴扎实推进，异地就医即时结算平台联网运行。养老体系建设不断加强，新增养老床位2760张。教育优先战略深入实施，新改扩建中小学校95所，达川、大竹义务教育均衡发展通过国家评估认定，达一中等示范高中改革创新全面推进，嘉祥学校扩大招生，西南职教园区加快建设。健康达州行动扎实开展，达州中医药职业技术学院成功纳入全省规划，与华西医院合作办医加快推进，分级诊疗成效持续巩固。食品药品监管切实加强，“毒血旺”“地沟油”等违法行为得到严惩，网络订餐+视频“明厨亮灶”试点开展。全民健身活动广泛开展，成功创建国家高水平体育后备人才基地。文化惠民工程深入实施，第六届全国新农村文化艺术展演圆满举办，市档案馆建成投用，罗家坝遗址博物馆启动建设，新改扩建农村综合文化服务中心308个。安全生产责任制有效落实，连续四年未发生重特大安全事故。平安达州建设深入推进，社会保持和谐稳定，为党的十九大和省第十一次党代会胜利召开营造了良好的社会环境。

（七）自身建设切实加强。自觉增强“四个意识”〔8〕，持续巩固“两学一做”〔9〕，认真学习贯彻党的十九大精神，坚决维护以习近平同志为核心的党中央权威，坚决服从市委领导，从严治党、从严治政制度规定有效落实。自觉接受市人大的法律监督、工作监督和市政协的民主监督，广泛听取并采纳各方意见，法治政府加快建设。“放管服”改革〔10〕深入推进，市本级行政许可和公共服务事项下放或取消67项，不合法不合理证明取消294项，“双随机一公开”〔11〕监管全覆盖，一体化政务服务平台与全省同步上线运行；全流程电子化交易、异地远程评标试点在全省率先开展，公共资源交易场内场外监督进一步加强，实现增收节支33亿元。严格执行中央八项规定精神，认真落实省、市委相关规定，廉政建设和反腐败工作扎实推进，治庸问责、电视问政深入开展，正风肃纪持续深化。

与此同时，我们统筹兼顾，积极支持全市各条战线开展工作，国防动员、人民防空、民兵预备役建设、双拥共建和优抚安置工作不断加强，妇女、儿童、老龄、残疾人、慈善、红十字会等事业全面发展，民族、宗教、对台、外事、侨务、文联、科协、统计、保密、气象、地方志等工作得到巩固提升。

各位代表！刚刚过去的一年，我们在宏观经济环境和达州基本市情尚未发生根本变化的条件下，面对稳增长、去产能、调结构、促转型、治环境、保稳定以及脱贫攻坚等多重艰巨繁重任务，能取得这样的成绩实属不易。这是省委、省政府和市委坚强领导的结果，是市人大、市政协和社会各界监督支持的结果，是全市广大干部群众团结一心、攻坚克难的结果！实践证明，市委提出的“12335”总体思路完全符合中央精神和省委要求，完全符合达州实际，必须认真领会并坚定不移地贯彻执行；市政府成建制、系统性研究出台的系列政策措施是科学的和行之有效的，必须持之以恒地推进落实。在此，我代表市人民政府，向全市人民，向各位人大代表、政协委员，向各民主党派、工商联、无党派人士和人民团体，向驻达解放军指战员、武警官兵和政法干警，向所有关心、支持和参与达州建设发展的社会各界朋友，表示衷心的感谢！

在肯定成绩的同时，我们也清醒地认识到，政府工作还存在一些不足，全市改革发展还面临不少困难和挑战，主要表现在：发展不平衡不充分问题尤为明显，基础设施欠账多，城乡区域发展差距较大，贫困人口量多面广；经济转型发展任重道远，传统产业占比偏高，新兴产业支撑不足，科技创新能力较弱；人居环境有待进一步改善，教育医疗、居住养老等公共服务保障和供给还不足，环境保护、城市管理、食品药品监管等与群众的期望还有较大差距；依法行政水平有待进一步提高，部分干部的担当、服务、法治、创新、危机等意识还不够强，少数公务人员不廉不勤等问题仍然存在。对此，我们一定高度重视，切实整改，不断开创政府工作新局面！

二、2018年目标任务

2018年，是全面贯彻党的十九大精神的开局之年，是我市巩固良好发展势头、确保经济持续增长、决胜同步全面小康、完成“十三五”规划的关键之年。今年政府工作的总体要求是：全面贯彻党的十九大精神，以习近平新时代中国特色社会主义思想为指导，认真落实中央和省委经济工作会议部署，按照市委四届五次全会暨经济工作会议要求，坚持稳中求进工作总基调，坚持新发展理念，始终保持加快发展、转型发展定力，提高发展质量、加快发展速度，坚定实施市委“三大战略”〔12〕，着力构建“五大新格局”〔13〕，以供给侧结构性改革为主线，统筹做好稳增长、促改革、调结构、提质量、惠民生、保稳定、防风险各项工作，确保全市经济社会持续快速健康发展。

今年发展的主要预期目标是：地区生产总值增长7.8%以上，固定资产投资增长9%以上，社会消费品零售总额增长11.5%以上，地方一般公共预算收入增长7.8%以上，城镇和农村居民人均可支配收入分别增长8 %、9%以上，居民消费价格涨幅控制在3%左右，城镇登记失业率控制在4.2%以内，全面完成省政府下达的脱贫攻坚、节能减排、生态环保目标任务。

各位代表！做好今年的政府工作，实现上述目标，我们将切实做到“四个坚定不移”。

坚定不移加快发展。发展不足、发展滞后仍然是达州的最大市情，加快发展始终是我们的第一要务。必须充分挖掘和发挥我市“交通、区位、人口、农业、能源、金融”六大比较优势，认真落实“全域精准脱贫攻坚、全域川陕革命老区、国家天然气综合开发利用示范区”三大特别政策，针对重点难点和关键点，以开展“工作年活动”为抓手，切实加大工作力度和强度，集中精力抓发展，千方百计做大经济总量，全力以赴做强经济质量。在实际工作中，我们将按照高于全省和全市预期目标来安排布置今年的各项工作。

坚定不移转型创新。我市经济结构性矛盾尤为突出，要实现持续健康发展，转型创新是我们必须迈过的坎。必须坚持质量第一、效益优先，大力开展制度创新、管理创新、技术创新，推动经济发展质量变革、效率变革、动力变革，提高全要素生产率，加快建设现代化经济体系。必须坚持一手抓传统产业存量的改革提升、一手抓新兴产业增量的培育发展，一手抓制造业的做大做强、一手抓现代服务业和现代农业的加快发展，着力推进产业结构、产品结构和投资结构转型升级，打牢经济持续健康发展基础。

坚定不移改革开放。改革开放是内陆和欠发达地区加快发展、追赶跨越的根本出路。必须以钉钉子的精神抓好国家和省各项改革措施的落实落地，以更加解放的思想、更大的决心深化各领域改革，切实解决制约我市发展的主要矛盾和矛盾的主要方面，最大限度地激发内生活力。必须牢固树立开明开放发展理念，主动融入全国全省开放发展大格局，大力营造最优的法治环境、政务环境、政策环境，着力打造最佳的投资洼地，依靠招商引资和对外合作来调整产业结构，借势借力推进发展。

坚定不移改善民生。改善民生是政府工作的出发点和落脚点。必须始终坚持以人民为中心的价值取向，努力解决好教育、医疗、环境、住房、养老、交通、食品药品安全等突出民生问题，着力打造宜居宜业环境，大力促进社会公平正义。必须坚定不移地把脱贫攻坚作为头等大事，精准发力、精准施策，确保全年目标任务的圆满完成。必须把民之所望作为施政所向，优先保障民生投入，优先安排民生项目，优先解决民生难题，让全市广大群众成为改革发展的最大受益者。

在思路和措施上，我们将做到“四个更加注重”，即更加注重在全国全省发展大局中去谋划区域发展，更加注重将达州发展置于成渝经济区和川东北区域发展中去定位，更加注重发展经济与保护生态环境、防范金融风险和改革开放创新相结合，更加注重在方法措施上集成集群集中以“组合拳”方式强力高效推进发展。

三、2018年重点工作

面对今年艰巨繁重的改革发展稳定任务，我们将围绕高质量发展，突出重点、统筹兼顾，着力抓好以下七个方面的工作。

（一）全力推进经济加快发展。坚持存量抓改革、增量抓创新，大力支持实体经济，分类施策做强县域经济，做好第四次全国经济普查工作，努力培育“四上一新”〔14〕企业，巩固“稳”的基础，积蓄“进”的力量，促进“质”的提升，不断扩大经济向好发展态势。

精准扩大有效投资。大力开展“项目攻坚突破年”活动，精准把握国家和省政策走向、投资方向，积极争取普惠性政策，全力争取特惠政策和项目支持，力争在项目储备、项目开工、项目建设、项目竣工等方面取得新的重大突破。引导资金更多投向补短板、调结构、促创新、提质量、惠民生等领域，市本级突出抓好30个重大项目，每个县（市、区）必须抓好10个以上重大项目，确保固定资产投资稳步增长。着力提高产业投资比重，完成工业投资280亿元以上、服务业投资800亿元以上。加大交通、水利等基础设施投资力度，加快弥补历史欠账。激发民间投资活力，再推出一批政府与社会资本合作项目，不断扩大民间投资规模。加强资金保障，创新融资方式，扩大直接融资成效。高度重视政府债务和金融风险防控工作。

大力实施工业强市战略。始终把发展工业作为经济工作的重中之重，认真开展“工业巩固提升年”活动，确保规上工业增加值增长8.7%以上。加快培育产业集群，抢抓建设国家天然气综合开发利用示范区的机遇，稳步推进天然气勘探开发，加快以瓮福为龙头的磷酸盐产业发展，着力打造能源化工产业集群；大力支持川环科技、新达泵阀、川东电缆、金恒机械等企业创新发展，加快云内动力专用车、清洁能源汽车等项目建设，推动机械汽车产业集群焕发生机、做大做强；抓好宣汉微玻纤产业的延链补链，加快玄武岩纤维产业园建设，尽快形成新材料产业集群；加快深圳卡立通手机及智能终端项目建设，抓好OPPO等知名企业配套的电子信息产业招商，市县共建川渝合作产业园，推动电子信息产业集群异军突起；引导我市医药企业与知名品牌企业兼并重组，扶持食品加工企业加快发展，不断壮大食品医药产业集群规模；加快引进361°等知名企业，支持南国印染、玉竹麻业等企业延链补链开发新产品，促进纺织服装产业集群形成新的增长极。力争全年工业重点项目开工30个、竣工投产30个。着力培育一批大企业大集团和新兴成长型企业，新增超十亿元企业2户、超亿元企业20户、规上企业50户。建好发展平台，加强园区污水处理厂等基础设施和服务平台建设，新增道路30公里、七通一平3400亩，新建标准厂房30万平方米；大力支持各类园区达标升级，创建省级以上经济开发区和高新技术产业园区。

加快发展现代服务业。狠抓服务业提档升级8个方面30条措施落地见效，确保服务业增加值增长9.5%以上。认真落实促进金融业发展“十条”意见，开工建设马踏洞“IFC”金融中心，着力推动达州银行、达州农商行“强行升位”，确保金融业增加值增长9%以上。持续推进秦巴物流园区、双龙公路铁路仓储区、仁和春天、复兴农产品批发市场、达川商贸物流园区等“十亿级”项目建设，做强服务业发展支撑。大力实施旅游业三年攻坚方案，突出抓好中心城区“两山两湖两岸”〔15〕、北部片区“一山一河一谷”〔16〕综合开发，启动建设八台山-龙潭河-巴山大峡谷、铁山大道等旅游通道，力争创建国家4A旅游景区2个，建成开放巴山大峡谷、牛卯坪等重点景区，推出一批达州自己的度假休闲和深度旅游景区，力争旅游总收入达到200亿元。加强市场调控和监管，确保房地产市场平稳健康发展。适应消费需求变化，完善政策措施，丰富消费供给，着力改善旅游、购物、居住、康养等消费环境，大力开展放心舒心消费城市创建，尽最大努力把消费留在达州，尽可能地吸引外地来达消费，不断做大消费总量。做大做强电商企业，鼓励达州名特产品电商化，切实解决“买多卖少”和“异地企业注册”等问题。

着力发展新兴产业。加快新型建筑建材业发展，开工建设建筑建材产业园，推进装配式建筑和新型建材快速发展，新增建筑资质企业20家，建筑业增加值增长10%以上。启动建设环保产业园，推动生活垃圾、餐厨垃圾、医疗废物等固废集中高效长效常态处理。加快引进和大力发展节能环保装备等绿色产业，支持企业开发绿色产品、建设绿色工厂、打造绿色供应链。大力培育发展新经济，推动大数据、物联网、云计算等新信息技术与实体经济深度融合，充分运用“互联网+”等创新创业新模式，形成一批涵盖三次产业的新业态。

（二）认真实施乡村振兴战略。农业农村是制约我市同步全面小康的最大短板。我们将把“三农”工作作为重中之重，大力开展“乡村振兴开局年”活动，着力打造一批新型乡村，让广大农村群众享受到现代文明生活。

加快现代农业发展。大力推进农业供给侧结构性改革，调优农业产品结构、供给结构和产业布局，提高农业综合效益和竞争力，确保农业增加值增长3.5%。全面落实粮食安全生产责任，确保粮食安全。突出建基地、夯基础、活机制三大重点，大力发展“3+6”〔17〕特色优势农业，创建省级现代农业产业融合示范园区2个，新建农建综合示范区21万亩，新培育省级示范农民合作社8个、示范家庭农场10家。加快补齐加工、贸易、农旅三大短板，重点抓好知名农产品加工和贸易企业招商，推动农产品加工和贸易实现突破性发展。开发农业多种功能，支持城市近郊以观光农业为依托发展乡村旅游、有条件的农村发展森林康养，多渠道增加农民收入。强化农业与工业、服务业的联动发展，积极构建三次产业互为补充、协同发力的产业发展新格局，推动农业资源优势向产业和经济优势、农业大市向农业强市转变。

加强幸福美丽乡村建设。坚持两条腿走路，对已建新农村聚居点、新农村综合体，按照乡村振兴要求进行完善提升；对新建易地扶贫搬迁集中安置点和新村，直接按照乡村振兴要求，采用脱贫攻坚引领区建设模式进行高标准打造。坚持园区、景区、产业基地与村庄相融，高起点规划、高质量建设一批特色小镇、幸福美丽新村和田园综合体，持续创建“四好村”，在规划、体制、机制、模式上不断探索创新，为推动乡村振兴起好步、开好局。加快建设“1+6”〔18〕在建大中型水库，完善宝石湖等渠系配套。加强农村基础设施和公共设施建设，新改扩建农村公路1000公里，改造农村土坯房3.3万户。开展农村环境整治行动，推进垃圾、污水、厕所 “三大革命”，着力改善农村人居环境。

深化农业农村各项改革。加快推进农村产权制度和农业经营体制改革，落实第二轮土地承包到期后再延长30年，推进承包地“三权”分置运行，完成农村产权确权颁证，完善农村产权交易市场和农业社会化服务体系，培育新型农业经营主体。健全自治、法治、德治相结合的乡村治理体系，支持农民工返乡创业，鼓励高校毕业生、科技人员等到农村施展才华，培养造就一支懂农业、爱农村、爱农民的“三农”工作队伍、农村乡贤和新型农民。

（三）加快推进新型城镇化。以国家新型城镇化综合试点为抓手，坚持以人为核心，强化互联互通，优化功能布局，打造宜居环境，提升管理水平，推动市域协调发展，确保户籍城镇化率提高1.3个百分点。

加快现代综合交通枢纽建设。围绕打造中国西部现代综合交通枢纽，全力争取开工建设成南达高铁，加快达州高铁站、西渝高铁前期工作，推动我市早日全面融入国家八横八纵高铁路网，形成十字交叉高铁枢纽。提速达州新机场、营达高速、巴万高速和达宣、达开、万源至八台快速通道等项目建设，加快镇达广、开梁、平宣开、城宣大高速和达渠、宣南快速通道等项目前期工作，开工市绕城高速西段、新机场航站区、G210线达州过境段三期等项目，统筹抓好国省干线改扩建和县乡道路建设，积极推进渠江航道升级改造，着力构建域内域外快速通畅的现代综合交通体系。

加强中心城市建设管理。围绕今年建成“双100”、2020年建成“双130”区域中心城市〔19〕，大力实施中心城区建设“三年攻坚计划”。加强城市规划，以城市空间格局发展研究成果为指引，启动城市总规前期修编，推进中心城区控规全覆盖。加强城市骨干道路建设，加快推进河市大道、机场大道等项目建设，推进罗江、徐家坝、野茅溪和中坝4座大桥建设，建成快速通道拓宽改造、环城路二期、凤凰山山前路、金南大道西延线Ⅰ期等项目，新建停车泊位1.5万个，从根本上缓解主城区交通拥堵问题。大力拓展城市空间，加快北城滨江、西城马踏洞和莲花湖、南城三里坪、翠屏山和长田坝等新区开发，着力推进塔沱、火车站、川鼓、南坝等片区棚改，引进一批知名开发企业入驻，提升城市建设品质。开展城市“双修”〔20〕，加强市政设施、公共服务设施建设，开工建设城市体育运动公园，建成大寨子、紫荆花等城市公园。大力开展“城市综合管理年”活动，坚持政府主导、部门主推、辖区主责、社会协同、公众参与，全面推进城市“公共交通、交通秩序及停车场、综合执法、环卫保洁、文明建设、社会治安”六大领域的精细化管理，确保城市管理水平大提升。

推进宜业宜居城镇建设。加强县城建设，重点抓好新区开发和产业新城建设，同步完善市政设施、公共服务设施，提升人口和产业承载能力，加快推进Ⅰ型、Ⅱ型小城市建设。积极开展大竹宜居县城、海绵城市试点，推进宣汉县多规合一试点。深入开展百镇建设行动，加快17个省级试点镇建设，因地制宜规划建设宣汉胡家、渠县龙潭等一批独具特色的小城镇，推进20个中心镇向新型小城市发展，培育省级特色小镇1个、试点镇3个。

（四）攻坚克难打赢脱贫攻坚硬仗。今年达川、大竹、渠县、开江4个贫困县要摘帽、全市319个贫困村要退出、15.0695万贫困人口要脱贫、4.6872万人要易地扶贫搬迁，脱贫攻坚任务尤为艰巨。我们将大力开展“精准脱贫决战年”活动，以“开局即决战、起步就冲刺”的姿态，全力攻坚克难。

加快脱贫项目建设。严格对照贫困县摘帽“一低三有”〔21〕、贫困村退出“一低五有”〔22〕、贫困户脱贫“一超六有”〔23〕要求，重点推进未达标的公共服务设施和基础设施建设，加快易地扶贫搬迁和建档立卡贫困户危房改造。突出早谋划早安排，上半年基本完成硬件建设，下半年抓好短板弥补和软件建设。持续开展脱贫攻坚引领区建设，统筹抓好新村、基础设施、公共服务、生态环境建设和产业发展，带动区域内贫困户与非贫困户共同发展，每个县（市、区）高质量新建1-2个引领区，构建区域脱贫连片发展新格局。

突出抓好产业扶贫。根据贫困村、贫困户的资源禀赋和条件，精准选择脱贫产业项目。加强涉农资金整合，用好四大扶贫基金，加快培育主导产业。大力开展电商扶贫，助推贫困地区农特产品走出大山。狠抓旅游扶贫，多渠道增加贫困群众就业和收入。加强金融扶贫，创新服务产品，加大“税农贷”推广力度。鼓励引导工商资本进入贫困地区，以农产品加工和贸易解决农村产业同质化问题，带动贫困地区产业大发展。坚持扶智与扶志相结合，加强技能培训和自力更生引导，确保符合条件的计划脱贫户发展1个增收产业或实现1人就业。

从严强化监督考核。严格开展验收考核评估，运用综合督查、专项检查，倒逼脱贫攻坚决策部署、工作责任、扶持措施、资金项目落到实处。严格驻村工作队伍管理，统筹抓好“5+2”帮扶工作，切实提高群众的认可度和满意度。深入实施扶贫领域突出问题专项整治行动，对违纪违规和工作执行不力、落实不到位的严肃追责问责。加强对已脱贫户、退出贫困村、摘帽县的跟踪检查，督促“回头看”“回头帮”有效落实，确保脱贫不返贫。

（五）深入推进改革开放创新。改革开放创新是加快发展的必由之路。我们将以更大的决心和力度推进改革开放创新不断向纵深发展，努力培育达州发展新优势。

全面深化重点改革。认真落实“三去一降一补”〔24〕重点任务，继续化解煤炭、水泥等行业落后产能，突出抓好商业用房去库存，落实减税降费政策，推动企业降本增效。深化行政管理体制机制改革，不断完善市区管理、城市执法管理、城市建管分离、国有资产管理等体制机制，持续优化行政效能、目标绩效考核。深化国有企业改革，实施“3+10”〔25〕国有资本布局调整战略，做实三大国有资本投资平台公司，做强10大集团企业，加快建立现代企业制度。深化投融资体制改革，发挥投资对优化供给结构的关键性作用。深化财政管理改革，狠抓财源培植，完善财经大数据平台，加强重点财源管控，确保税收占财政收入的比重稳步提高；创新政府配置资源方式，加强资金筹措使用统筹管理，改革财政支持方式，努力提高财政资金的使用效益。

纵深推进开放合作。大力开展“招商引资落地年”活动，狠抓项目包装储备、精准对接、签约落地等关键环节，突出政策优先，实施产业成链、集群抱团、国资结伴等招商策略，瞄准国内500强和行业100强，用好西博会、渝洽会等省内外重大投资促进平台，精心办好中外知名企业达州行、产业专题招商等活动，巩固形成“春夏秋”集中招商攻势，确保“6+2”产业集群专题招商每个方面都有一批重大项目集中或集群落地建设投产，新引进国内500强企业5家以上，引进到位资金800亿元以上，制造业招商项目占比达50%以上。推进全方位开放合作，主动对接国家“一带一路”和长江经济带战略，加快融入全国全省对外开放大格局，高起点承接产业转移；推进发展注册经济；继续加强区域合作、企地合作、校地合作、银政企合作。大力发展对外贸易，支持企业参加全省“万企出国门”活动，拓展海外市场，推动“达州造”优势产品走出去；加强出口企业和产品培育，抓好经开区磷硫化工、大竹鞋服、宣汉新材料等园区和特色农产品出口基地建设，力争进出口总额突破6000万美元。

大力推进创新发展。切实增强创新创造意识，充分尊重客观规律，大力开展体制创新、机制创新、管理创新、模式创新、技术创新和工作方式方法创新，把创新贯穿于各项工作之中，用创新创造引领和推动发展。落实科技创新“十个一批”〔26〕举措，引导企业加大投入开发新技术、新工艺、新产品，实施一批重大科技攻关项目，创建市级以上创新平台8家，培育国家高新技术企业3家，力争科技对经济增长的贡献率达到55%。全面落实鼓励创新创业的各项优惠措施，大力实施青年创业促进计划、大学生创业引领计划和巾帼创业行动。

（六）切实加强生态环境建设。牢固树立绿水青山就是金山银山的理念，大力开展“环境治理攻坚年”活动，巩固深化中央和省环保督察成果，努力让达州的天更蓝、水更清、空气更清新。

强化污染防治。全力推进中央和省环保督察发现问题的整改，确保按时按质全面完成。加快弥补环保基础设施短板，全面启动污水和垃圾处理PPP项目，建成市第二污水处理厂，抓好中心城区第二饮用水源前期工作。加强大气、水污染防治，认真落实“河长制”，深入开展城市扬尘、工业废气、燃煤锅炉等专项整治和州河、巴河、渠江等流域污染综合整治，突出抓好畜禽养殖污染、城市黑臭水体和“散乱污”企业治理，严格规范管理生猪屠宰、餐饮、建筑施工等行业，确保空气质量达标率持续上升、出境断面水质持续向好。加强土壤污染防治，防控土壤环境风险。推进环保管理体制改革。开展第二次污染源普查。加强环境监管执法，着力构建环境保护长效机制，始终保持严厉打击环境违法行为的高压态势。

加强生态建设。严守生态保护红线，加强森林资源和生物多样性保护利用，积极推进天然林保护区、自然保护区、森林公园、地质公园、湿地公园等保护建设。开展绿化达州行动，加快创建国家森林城市和园林城市，实施重点生态景观整治和修复，抓好城市公园、公共绿地和“沿路、沿河、沿库”生态廊道建设，推进天保二期工程、新一轮退耕还林建设，完成营造林30万亩，新增森林5000公顷，森林覆盖率达到43%。加强水土保持，强化地质灾害防治，加大生态脆弱区域和灾毁区域生态修复力度，治理水土流失150平方公里。

推进节能减排。加强产业项目全过程环保监管，实施工业污染源全面达标排放计划，削减污染物排放总量。推进工业废气、废水、废物的综合治理和回收再利用，开展企业清洁生产和重点产业园区循环化改造。抓好工业、交通、建筑等重点领域节能降耗，大力淘汰落后产能和工艺设备，倒逼“两高”行业转型升级。倡导和鼓励绿色生活方式，促进绿色消费、绿色出行、绿色居住。

（七）全面发展民生社会事业。让人民群众过上美好生活是政府始终不渝的奋斗目标。我们将大力开展“民生改善实效年”活动，努力让全市人民过上更好的生活。

提高民生保障水平。继续抓好中心城区十大民生实事，扎实推进九项民生工程，着力办好民生大事，确保财政民生支出不低于70%。认真落实就业创业政策，城镇新增就业3.4万人。大力实施全民参保计划，加强“五险”统征，巩固城乡居民医保制度，提升社会保障水平。健全重大疾病救助体系，清理规范城乡低保。大力发展民办养老、社区和居家养老服务，推进医养结合，促进养老服务提档升级。大力发展老龄、残疾人、慈善、红十字会等事业，加强农村留守儿童、妇女、老人关爱服务。

提升公共服务能力。大力发展教育事业，突出抓好义务教育均衡发展，新改扩建中小学校90所，确保宣汉、万源、渠县、开江通过国家义务教育均衡发展评估验收；市本级和每个县（市、区）集中精力办强一所公办示范高中；积极引进省内外优质教育资源落户达州；加快西南职教园区建设，启动主城区中职学校统筹整合和退城进园；大力发展学前教育；支持继续教育发展；推进四川文理学院转型发展成应用型大学、达州职业技术学院创建优质专科高职院校。深入实施健康达州行动，加强重大传染病防治，完善分级诊疗制度，推进公立医院改革创新，抓好华西医院达州分院、达州中医药职业技术学院建设，不断提升达州医疗健康水准。大力发展体育健身运动，着力增强人民体质。深入实施文化惠民工程，加快公共文化服务设施建设，加强巴文化保护和传承，鼓励文艺创作，推动文化事业和文化产业繁荣发展。认真做好民族、宗教、统计、气象、保密、档案、地方志、对台、外事、侨务等工作，促进社会全面进步。

创新推进社会治理。坚持专项治理、系统治理、综合治理、依法治理、源头治理相结合，推动政府治理、社会自我调节、居民自治良性互动。创新社会治理方式，引导社会组织广泛参与社区公共事务，促进社区、社会组织、社会工作者联动。健全重大决策社会稳定风险评估机制，完善矛盾纠纷多元化解机制，依法分类处理信访诉求。构建覆盖城乡的公共法律服务体系，持续开展“法律七进”〔27〕，做好法律援助、社区矫正和工读矫治工作。健全社会信用体系，加强政务、商务、社会诚信和司法公信建设。深化平安达州建设，加强治安乱点整治，依法严厉打击“黑拐骗”“黄赌毒”等各类违法犯罪，着力解决网上虚假信息诈骗、倒卖个人信息等突出问题；严格落实安全生产责任制，坚决遏制重特大事故发生；实施最严格的食品药品监管，全力整治“地沟油”，加大食品和农产品“双安双创”〔28〕力度，确保人民“舌尖上的安全”。加强国防动员、国防教育和国防后备力量建设，积极做好双拥共建、人民防空等工作，巩固军政军民团结。

四、加强政府自身建设

各位代表！政府自身建设永远在路上，面对新时代新任务，我们将以更高的标准、更严的要求、更实的作风，不断进行自我完善、自我革新和自我提高，持之以恒建设人民满意政府。

（一）坚定政治方向。深入学习贯彻党的十九大精神，自觉运用习近平新时代中国特色社会主义思想武装头脑、指导实践。牢固树立“四个意识”，严守党的政治纪律和政治规矩，坚定维护以习近平同志为核心的党中央权威，坚决贯彻执行党的路线方针政策，认真落实省委省政府的决策部署，坚决服从市委的领导，始终与市委同心同德、同向发力。切实加强政府班子思想政治建设，教育政府系统广大干部不忘初心、牢记使命。

（二）严格依法行政。坚决维护宪法法律权威，依法规范行使职权，自觉运用法治思维和法治方式推动工作。依法做好政府立法工作，积极开展综合行政执法改革，加强权力行使监管，全面推进法治政府建设。主动接受市人大、市政协监督，加强与民主党派、工商联、无党派人士的联系，重视司法、舆论和公众监督，广泛听取和吸纳各方意见。全面推进政务公开工作，让权力公开透明、阳光运行。

（三）持续转变作风。扎实开展“不忘初心、牢记使命”主题教育，坚决整肃庸政懒政怠政行为，严格落实工作岗位责任制，确保各项工作落实落地。纵深推进“放管服”改革，加快政府职能转变，着力提升行政效能和服务水平。用好容错纠错机制，旗帜鲜明为担当者担当，以担当担责精神应对新挑战、争创新业绩。大力弘扬坚韧不拔、不胜不休的作风，始终保持逢山开路、遇河架桥的精神状态。大力精简会议，原则将每月第一周确定为无会周，集中精力狠抓工作落实。

（四）提升能力水平。加强对新理论新政策新知识的学习，把握新时代改革发展的特点和规律，大力提升理论政策水平。整体考虑，系统谋划，精准施策，着力提升驾驭全局和处理复杂问题的能力。遵循经济规律、社会规律、自然规律，遵守程序规定，努力提升科学决策能力。解放思想，探索新机制，研究新方法，创造新举措，解决新问题，切实提升创新突破能力。

（五）加强廉政建设。始终把廉洁从政贯穿始终，坚决贯彻中央八项规定精神和省、市委相关规定，严格执行《准则》《条例》，全面履行党风廉政建设主体责任和“一岗双责”。深化廉政风险防控，加强政务监察和审计监督，狠抓重点领域、关键环节和重要岗位监督管理，坚决纠正和查处各种顶风违纪行为，努力打造一支忠诚、干净、担当的公务员队伍，让人民群众从政府各项工作中感受到清风正气。

各位代表！使命呼唤担当，实干成就梦想。我们将更加紧密地团结在以习近平同志为核心的党中央周围，在省委省政府和市委的坚强领导下，开拓进取，扎实工作，不断开创新时代达州改革发展的新局面，为实现同步全面小康、建成幸福美丽达州而努力奋斗！
